# Endoscopic ultrasound-guided drainage of a complex kidney abscess penetrating to the gallbladder and right liver lobe

**DOI:** 10.1055/a-2186-3511

**Published:** 2023-10-24

**Authors:** Katarzyna M. Pawlak, Mateusz Jagielski, Eryk Bella, Kareem Khalaf, Klaus Mönkemüller, Marek Jackowski

**Affiliations:** 1 The Center for Therapeutic Endoscopy and Endoscopic Oncology, St. Michaelʼs Hospital, University of Toronto, Toronto, Canada; 2 Department of General, Gastroenterological and Oncological Surgery, Collegium Medicum, Nicolaus Copernicus University in Toruń, Poland; 3 Department of Gastroenterology, Virginia Tech Carilion School of Medicine, Roanoke, Virginia, USA

A 64-year-old woman with a past medical history of poorly controlled diabetes, morbid obesity, hypertension, and status post-oncological treatment for metastatic sigmoid colon cancer was admitted to the surgery department due to a 50 × 40 × 43-mm abscess in the right kidney on computed tomography (CT) ( Fig. 1 ). The abscess penetrated the gallbladder, causing its perforation. It also penetrated the liver and was causing renal artery infiltration with pseudoaneurysm formation (21 mm and 16 mm in diameter) ( Fig. 2 ). The patient was clinically and biochemically septic. CT ruled out other sites of abscess. Due to significant associated co-morbidities, poor general status, and poor percutaneous access, neither a standard surgical intervention nor percutaneous drainage was feasible.

**Fig. 1:**
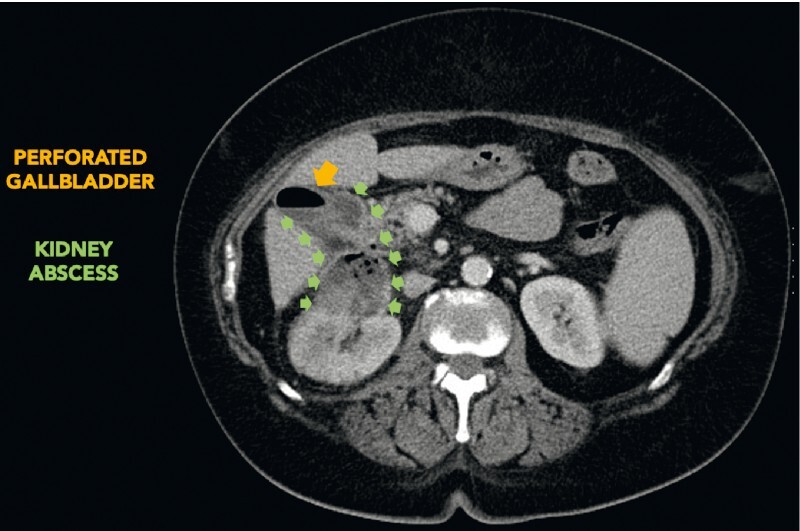
The 50 × 40 × 43-mm abscess in the right kidney, penetrating into the gallbladder causing its perforation.

**Fig. 2:**
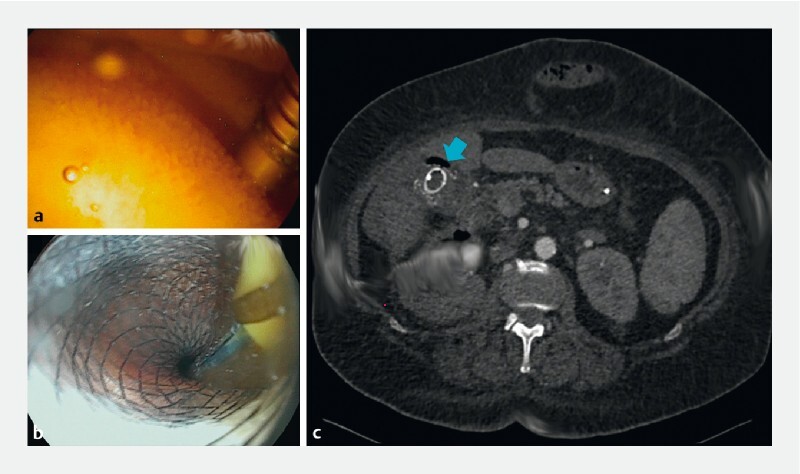
Endoscopic ultrasound-guided lumen-apposing metal stent (20 mm × 16 mm) placement for left kidney drainage through the gallbladder, followed by nasogastric tube placement.

As there was a connection between the kidney abscess and perforated gallbladder, endoscopic ultrasound (EUS)-guided drainage was performed under general anesthesia using a linear echoendoscope ( Video 1 ). Through transduodenal access after endosonographic visualization of the gallbladder, fine-needle aspiration (FNA) with a 19G FNA needle was performed. The aspired purulent content mixed with bile of the gallbladder confirmed the position. A 0.035-inch guidewire was advanced through the needle and looped in the abscess cavity under X-ray control. Then, the tract was dilated with a 10-Fr cystotome, followed by placement of a 20  ×  16-mm cautery-enhanced lumen-apposing metal stent (LAMS). Immediately, purulent content outflow was observed from the stent lumen. The procedure was concluded with placement of a 7-Fr nasogastric tube through the LAMS lumen for active abscess lavage (50 ml of saline every 6 hours).

**Video 1** Endoscopic ultrasound-guided drainage of a complex kidney abscess through perforated gallbladder using lumen-apposing metal stent with double pigtail.


After 3 weeks of active transmural drainage, symptoms resolved and laboratory parameters normalized. CT showed a collection decrease to 3 mm, given that the nasogastric tube was replaced with a 7-Fr × 7-cm double-pigtail stent. The patient was discharged and followed up in outpatient settings. After 5 months, CT showed a collection size of 21 × 10 mm (decrease> 50 %) and a healed gallbladder wall during follow-up endoscopic assessment ( Fig. 3 ). One year after the procedure, the patient remains asymptomatic, without collection, on LAMS stent and permanent double-pigtail drainage to prevent recurrence ( Fig. 4 ).

**Fig. 3:**
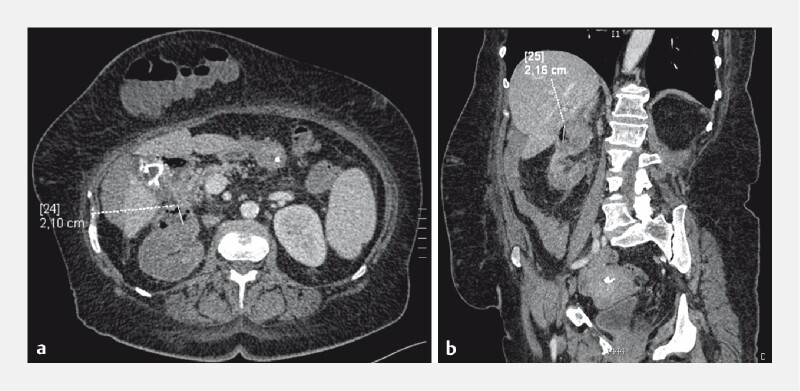
Computed tomography performed 5 months after the procedure: successful endoscopic treatment (collection size 21 × 10 mm; decrement > 50 %).

**Fig. 4:**
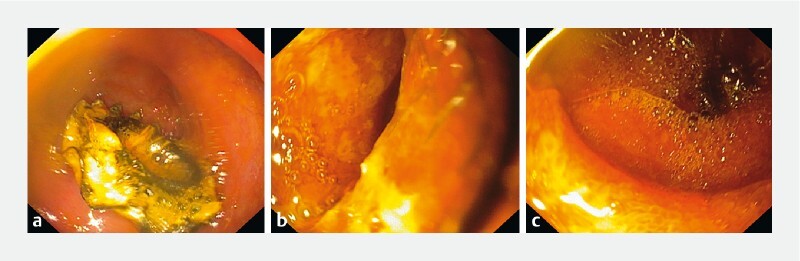
Surveillance esophagogastroduodenoscopy 12 months after the procedure: healed gallbladder wall; patient remains asymptomatic.

This case is important for three reasons. First, we show how a complex kidney abscess penetrating the gallbladder and liver can be successfully managed endoscopically. Second, EUS-guided drainage of a peritoneal cavity abscess might be an optimal alternative therapy for patients with multiple comorbidities, at high risk for life-threatening recurrent sepsis, and are poor surgical candidates. Lastly, similar to the management of other types of recurrent collections, long-term transmural drainage with an indwelling double-pigtail plastic stent seems to be an option for this group of patients 1 .

Endoscopy_UCTN_Code_CCL_1AF_2AZ_3AD

## Notes

Endoscopy E-Videos

https://eref.thieme.de/e-videos
*E-Videos* is an open access online section of the journal *Endoscopy* , reporting on interesting cases and new techniques in gastroenterological endoscopy. All papers include a high-quality video and are published with a Creative Commons CC-BY license. Endoscopy E-Videos qualify for HINARI discounts and waivers and eligibility is automatically checked during the submission process. We grant 100% waivers to articles whose corresponding authors are based in Group A countries and 50% waivers to those who are based in Group B countries as classified by Research4Life (see: https://www.research4life.org/access/eligibility/ ). This section has its own submission website at https://mc.manuscriptcentral.com/e-videos
